# The use of a modified abbé island flap to reconstruct primary lip defects of over 80 %

**DOI:** 10.1186/s40463-016-0148-0

**Published:** 2016-05-31

**Authors:** Sabin Filimon, Keith Richardson, Michael P. Hier, Michael Roskies, Alex M. Mlynarek

**Affiliations:** McGill University, Faculty of Medicine, McIntyre Medical Building, 3655 Sir William Osler, Montreal, Quebec H3G 1Y6 Canada; Department of Otolaryngology – Head and Neck Surgery, McGill University, Royal Victoria Hospital, 1001 Decarie Boulevard, Montreal, Quebec H4A 3 J1 Canada; Department of Otolaryngology – Head and Neck Surgery, McGill University, Jewish General Hospital, 3755 Cote-Sainte-Catherine street, Montreal, Quebec H3T 1E2 Canada

**Keywords:** Lip reconstruction, Abbé flap, Modified Karapandzic, Over 80 % lip defects

## Abstract

**Background:**

Lip reconstruction for defects greater than 80 % present a challenge in maintaining acceptable oral function and good aesthetic results. Abbé flaps offer an excellent reconstructive option but are limited to defects under 65 %.

**Methods:**

We describe a two-stage “modified Abbé island flap” technique whereby a full-thickness myocutaneous flap is combined with a modified Karapandzic flap, allowing for reconstruction of total and near total lip defects.

**Results:**

Six patients underwent successful two-stage lower and upper lip reconstruction with this technique. Oral competence and satisfactory aesthetic outcomes were achieved in all six cases. There were no complications. Although microstomia was noted to a certain extent, we argue this impact to be less than the morbidity of a free flap that lacks sphincteric function.

**Conclusion:**

The “Modified Abbé Island Flap” can be used to reconstruct near-total lip defects using locally innervated, well-vascularized tissues that recreate the oral sphincter and restore oral competence. The combination of the conventional Abbé flap with a modified Karapandzic flap provides reliable results and significantly reduces operating time.

## Introduction

**Fig. 1:**
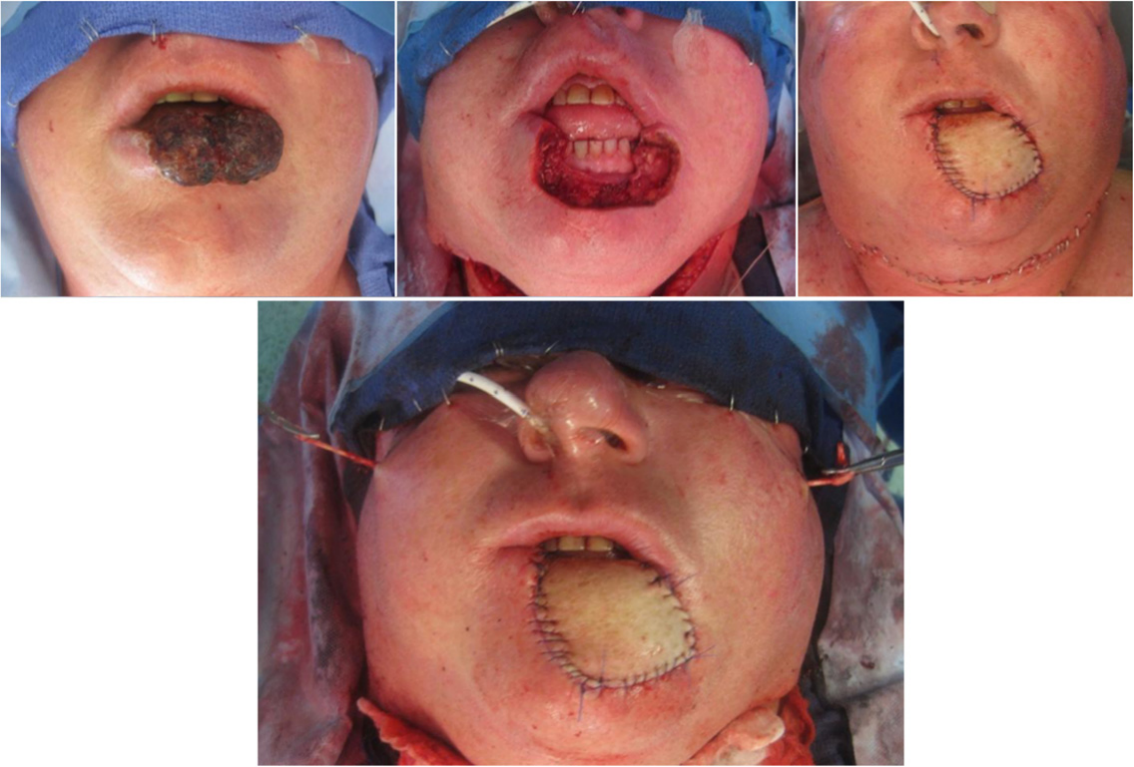
Radial forearm flap and palmaris longus tendon sling for reconstruction of a large squamous cell carcinoma of the lower lip. The palmaris longus is tunneled to an opening near the zygoma

Lip reconstruction for defects greater than 80 % present a challenge in maintaining acceptable oral function and good aesthetic results. Before the advent of free-tissue transfer, large lip defects of over two-thirds the length of the lip were reconstructed using bilateral Gillies or Karapandzic flaps [1] which resulted in cheek tissue de-enervation, compromised oral competence, significant microstomia, and inferior aesthetic outcomes. While the radial forearm free flap is a workhorse for head and neck reconstruction (Fig. 1), its use for total lip reconstruction is limited to poor functional outcomes, suboptimal aesthetic results, increased operative times, and donor site morbidity. Head and neck reconstructive surgeons are aware that, whenever possible, lip defects should be repaired using adjacent tissue. First described by Sabatini in 1837 [2], the Abbé flap has proven very versatile in the reconstruction of small to medium lip defects, limited in its usage only by defects larger than 65 %. The neurovascular full-thickness pedicled flap has the advantage of preserving the circumferential orbicularis oris muscles as well as the vermilion border while attaining good aesthetic results without loss of sensation. Additionally, minimal donor site morbidity is observed and operating times are significantly decreased.

Using these same principles, recent experience at our institution proves that it is possible to reconstruct lip defects of over 80 % following surgical resection of primary lip carcinoma by using a combination of neurovascular rotation and advancement flaps. We describe herein six-lip reconstruction cases performed successfully using a combined Abbé and modified Karapandzic flap. A detailed description of the surgical procedure, discussing the versatility of the new technique and its advantages over the original method, is presented.

## Material and methods

### Patients

Six patients (4 males and 2 females), ranging from 53 to 84 years of age, underwent primary surgical resection of upper and lower lip tumors over a four-year period. The clinical findings of the six-presented cases are summarized in Table 1. The combination Abbé and modified Karapandzic flap was used for closure in all cases after a full thickness resection was performed. In four out of six cases, the Karapandzic flap was done through a musculo-mucosal advancement to avoid further skin scaring. The maximal tissue borrowed from the donor lip was 40 %. A radial forearm – palmaris longus tendon free flap - was prepped at the onset in the event that our resection was deemed to be too extensive.

**Table 1:** Summary of clinical findings

Clinical finding	Case 1	Case 2	Case 3	Case 4	Case 5	Case 6	Ratio and mean
Age (years)	67	84	56	72	67	53	66.5
Sex	M	F	M	F	M	M	2:1
Smoking History	Yes	Yes	Yes	Yes	Yes	Yes	6/6
Tumor^a^	BCC	MCC	KA	SCC	SCC	SCC	-
Upper/lower lip	Upper	Upper	Lower	Lower	Lower	Lower	2/4
Stage	T3N0M0	T3N0M0	N/A	T3N0M0	T2N0M0	T2N0M0	-
Defect of the lip	85 %	90 %	90 %	85 %	85 %	85 %	86.6 %

^a^ *BCC* Basal Cell Carcinoma, *MCC* Merkel Cell Carcinoma, *KA* Keratoacanthoma, *SCC* Squamous Cell Carcinoma

Post operative management after the initial stage of the flap was as follows: the patients were started on liquids, purée foods and pain medicine with oral intake through a straw. Petroleum jelly was applied on the incision daily and patients were able to begin cleaning the incision on postoperative day five. Patients returned at seven days for suture removal and at four weeks for second-stage pedicle division. No other post-operative protocol, speech language therapy or lip stretching was used.

Patients were followed-up every two months for the first year, four months for the next two years and once a year up to five years post-surgery. The final functional and aesthetic outcomes were measured by using a special form developed in our clinic and completed for each patient at least one year post surgery (Table 2). To reduce observer bias, a protocol for recoding the results was put in place. The patients were seen at the same visit by two otolaryngologists uninvolved in the surgeries. The reported numbers represent the score given by each clinician separately. No disagreements were recorded.

**Table 2:** The form used in measurement of functional and aesthetic results

	Scores	Case 1	Case 2	Case 3	Case 4	Results
Functional assessment						
Sensibility						
- Hypoesthesia	1					
- Normal	2	2	2	2	2	100 %
Competence						
- Incompetence	1					
- Sialorrhea at rest	2					
- Sialorrhea with fluid	3	3	3			50 %
- Complete competence	4			4	4	50 %
Speech						
- Not intelligible	1					
- Partial intelligible	2					
- Fully intelligible	3	3	3	3	3	100 %
Preservation of facial expression						
- Asymmetric	1					
- Symmetric	2	2	2	2	2	100 %
Dietary and cutlery usage						
- Diet and cutlery modified	1					
- Eats as before surgery	2	2	2	2	2	100 %
Aesthetic Assessment						
Stoma						
- Severe microstomia	1					
- Moderate Microstomia	2					
- Mild Microstomia	3	3	3	3	3	100 %
Symmetry of commissures						
- Not symmetrical	1					
- Symmetrical at rest	2			2	2	50 %
- Symmetrical at rest and mouth opening	3	3	3			50 %
Amount of Scaring						
- Visible from distance	1					
- Visible at proximity	2		2			25 %
- Visible face to face	3	3		3	3	75 %
Preservation of the philtrum						
- Philtrum absent	1			1		25 %
- Philtrum present	2	2	2		2	75 %
Overall satisfaction						
- Not satisfied	1					
- Somewhat satisfied	2					
- Satisfied	3	3	3	3		75 %
- Very satisfied	4				4	25 %

The functional assessment included evaluation of lip sensitivity, which was considered normal if patients could feel a pinprick and light touching. Oral competence was differentiated into incompetence, sialorrhea at rest, sialorrhea with fluid intake, and complete if the patient was able to squirt liquid from the mouth. Speech intelligibility, preservation of facial expressions (such as smiling, kissing, pouting the lips, etc.), and diet and cutlery usage were also assessed. Aesthetic assessment included measurement of microstomia, which was considered mild if the mouth opening was sufficient to make possible denture insertion, the amount of scaring and preservation of the philtrum. Finally, comparison of the symmetry of commissures was also included. The form ended with the overall satisfaction of the patients with the results.

### Surgical technique

**Fig. 2:**
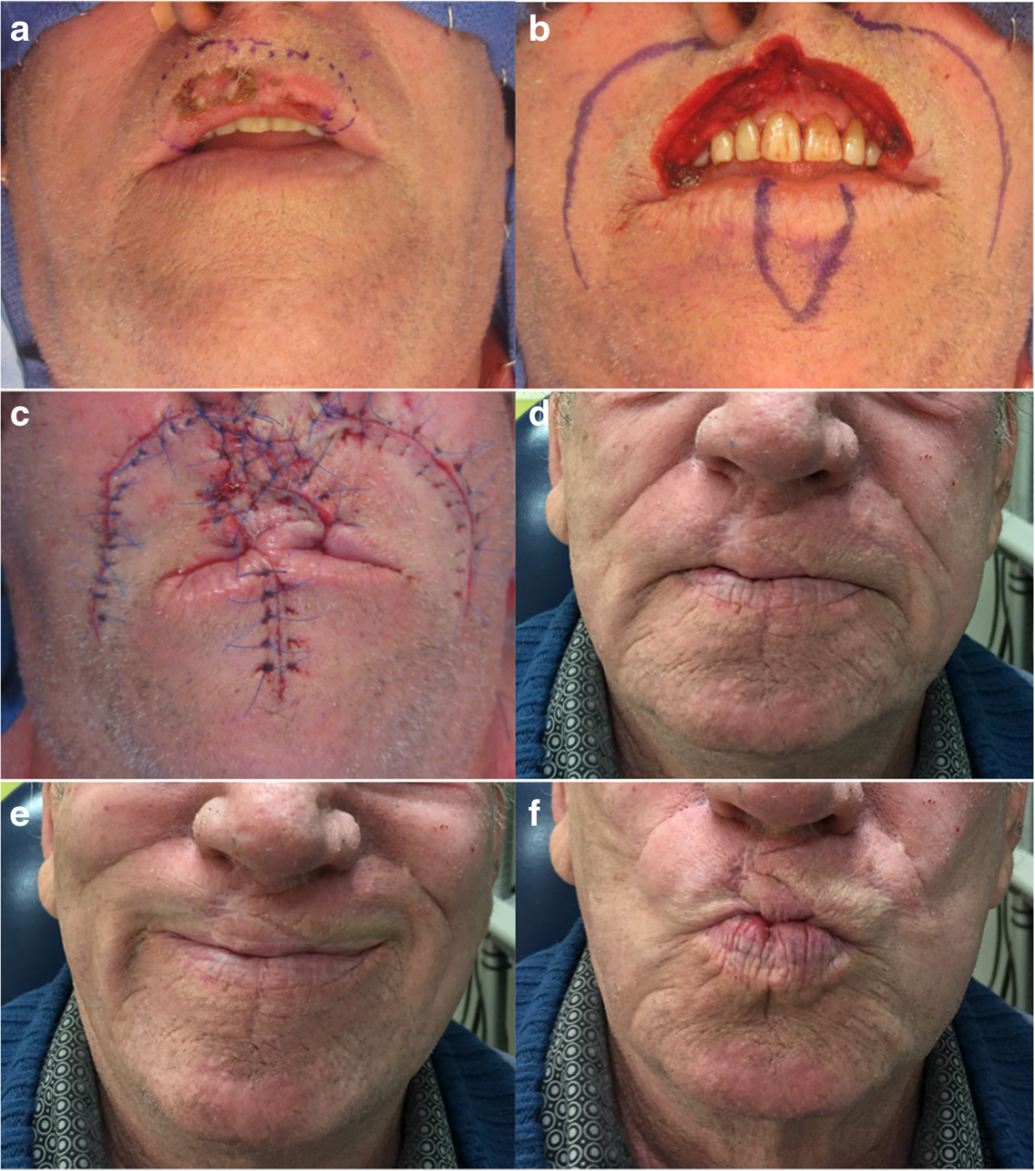
Case 1. **a** Large Basal cell carcinoma involving the upper lip. **b** Resection of BCC with 1 cm margin. **c** Abbé plus bilateral Karapandzic flaps sutured in place. (**d**) (**e**) (**f**) One-year post-operative results with good mouth opening and oral competence

**Fig. 3:**
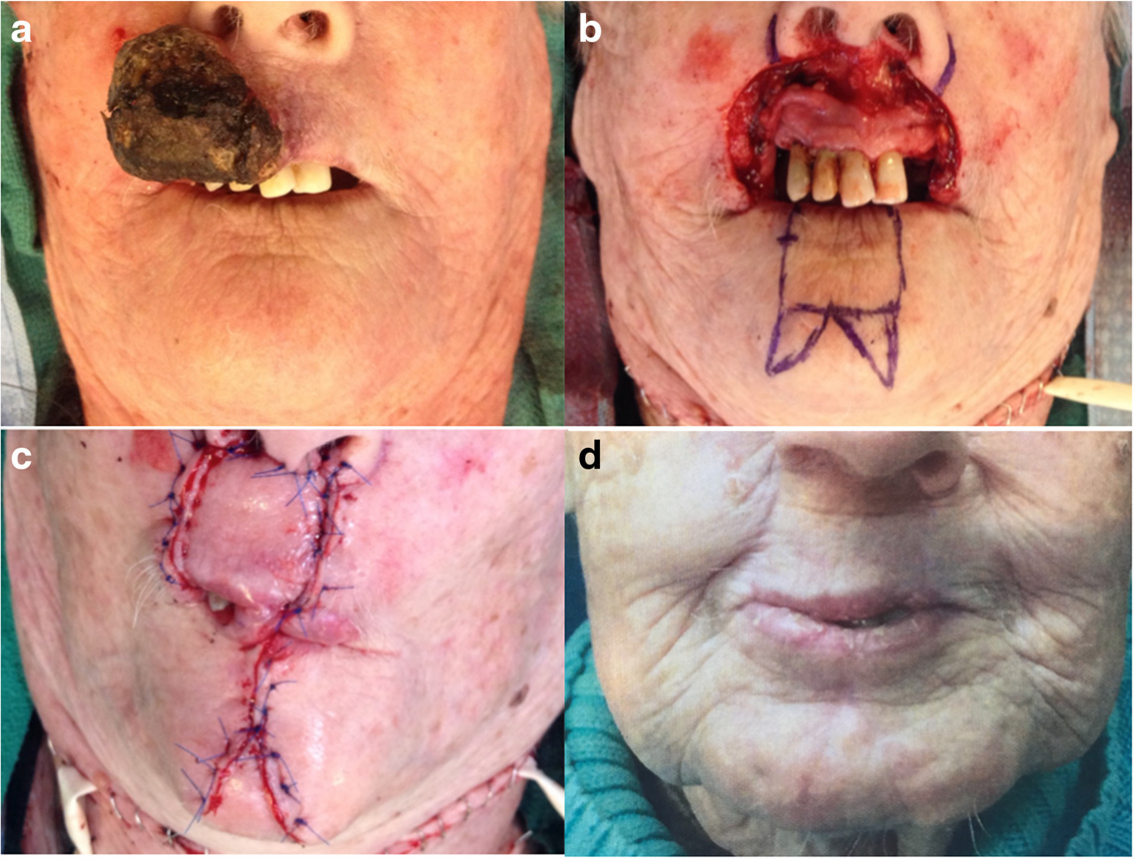
Case 2. **a** Large Merkel cell carcinoma involving most of the upper lip. **b** Resection of the tumor with 1 cm margin. **c** Abbé flap sutured in place with primary closure of the donor site. **d** One-year and a half postoperative results with good cosmetic and functional outcomes

**Fig. 4:**
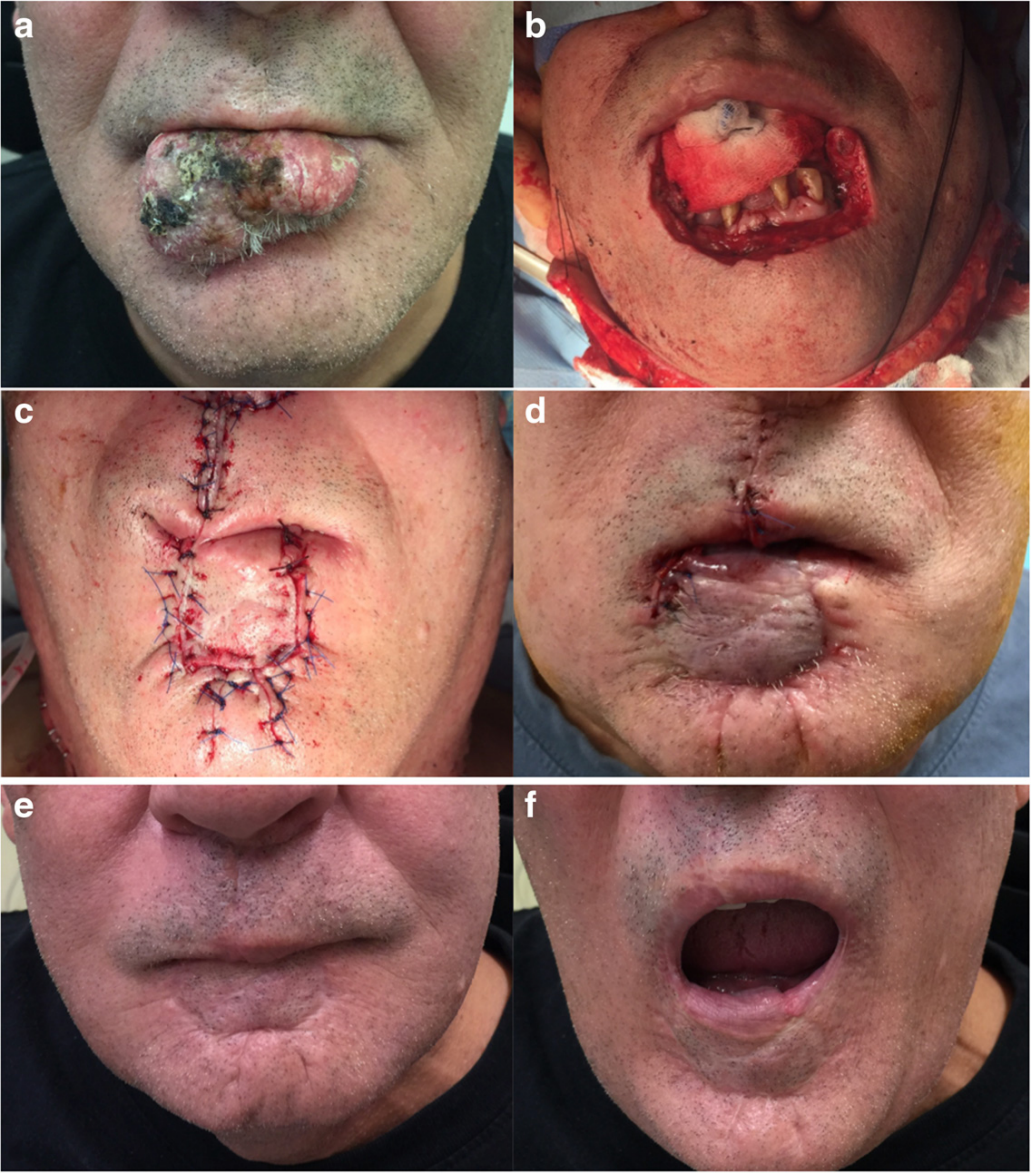
Case 3. **a** Large Keratoacanthoma involving the lower lip. **b** Resection of the tumor with 1 cm margin. **c** Abbé flap sutured in place with primary closure of the donor site. **d** Division of the pedicle flap at six weeks. (**e**) (**f**) One-year postoperative results with good cosmetic results and normal mouth opening

Under general endotracheal anesthesia, the area surrounding the tumor was infiltrated with 1 % lidocaine with 1:160 000 epinephrine. Lesions were resected with at least one-centimeter margins (Fig. 2a) as confirmed by intra-operative frozen section analysis. After complete tumor resection, the reconstruction was initiated by designing the Abbé flap on the unaffected lip so that the length of the flap approximated the length of the defect (Fig. 2b), providing coverage to the base of the columella in some cases (Fig. 3c). The flap was designed in a triangular (Fig. 2b) or W shape (Fig. 3b) according to the size of the defect. A full-thickness incision through the skin, muscle, and mucosa was made leaving the neurovascular structures intact. Further tissue loss was addressed by mobilization of cheek tissue through a modified Karapandzic flap. A curvilinear incision was made through the skin on both sides of the lip (Fig. 4 c), beginning on the inferior border of the surgical defect or lower edge of the Abbé flap and elongated by the mentolabial and nasolabial creases. In four out of the six cases, to avoid further facial scaring, the Karapandzic flap was performed using a musculo-mucosal advancement instead of a full thickness one (as see in Figs. 3c and 4c where the curvilinear incisions usually made in the nasolabial creases are absent). Branches of the facial nerve and labial artery were dissected and preserved to ensure innervation and perfusion. Suspensory muscles were lysed selectively until the cheek segments could be advanced to permit union with the Abbé flap. The union of the two flaps allowed for the creation of a new sphincter that was then sutured to the remaining muscle. The closure was performed in three successive layers, ensuring proper restoration of the orbicularis oris sphincter. The donor site was then closed primarily. After four weeks, the pedicle was divided in a clinic setting under local anesthesia and the inset of the flap was completed (Fig. 4d).

## Results

**Fig. 5:**
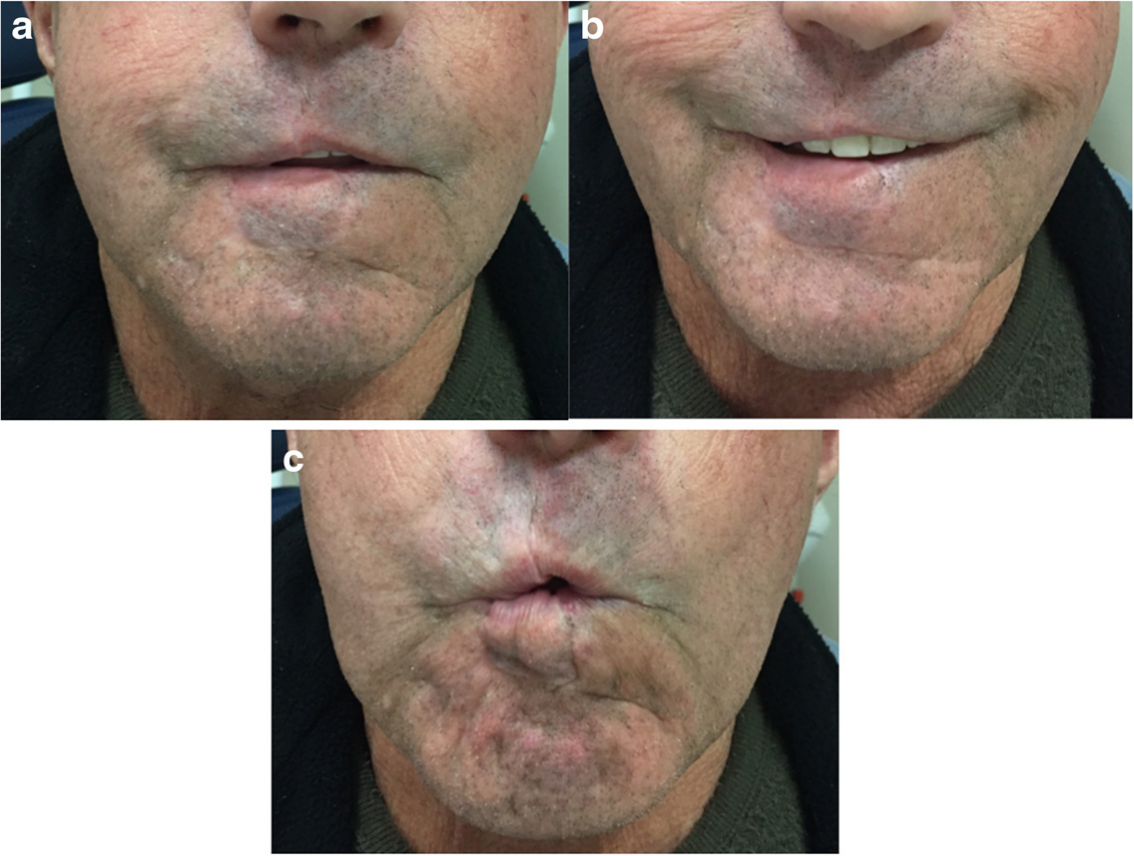
Case 5. (**a**) (**b**) (**c**) One-year postoperative pictures with good lip function and aesthetic results

**Fig. 6:**
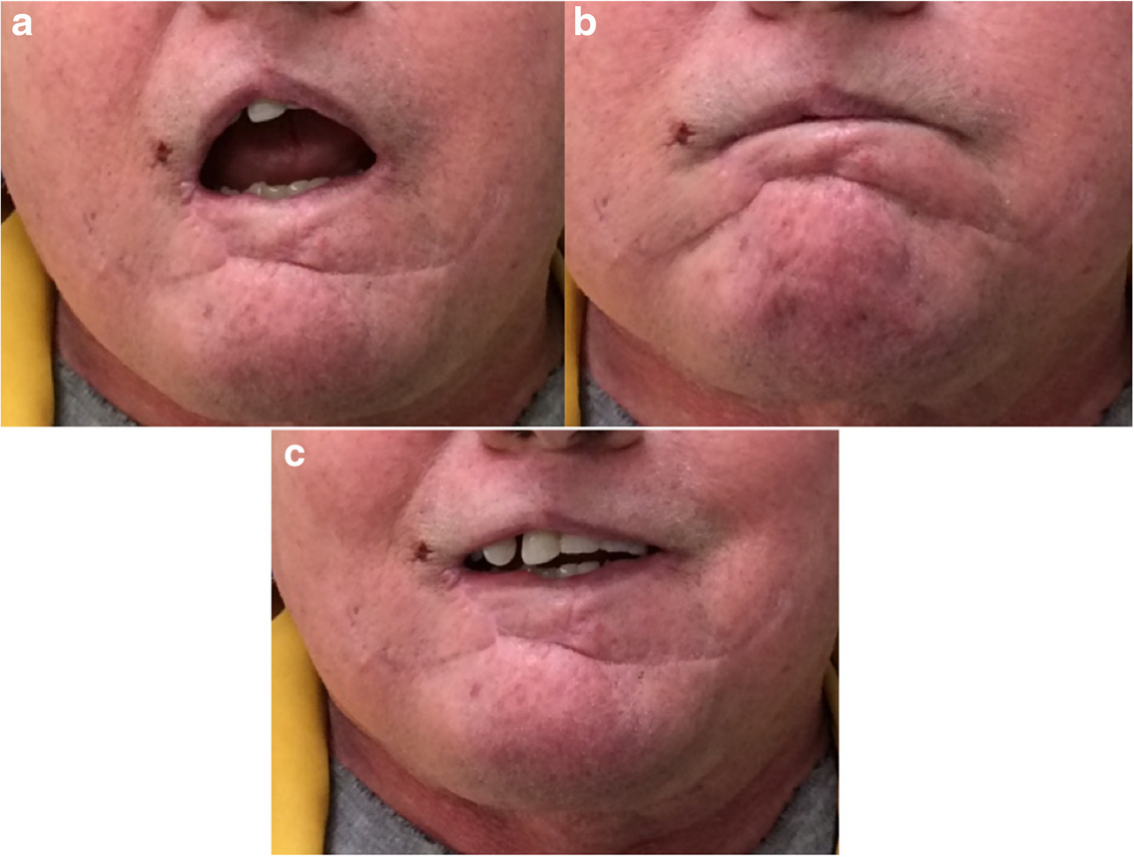
Case 6. (**a**) (**b**) (**c**) One-year postoperative pictures with good lip function and aesthetic results

After surgical resection, the resulting defect averaged 86.6 % of the total lip length (Table 1). The oral commissures were not involved in any of the cases. All flaps remained viable until division without venous congestion. Possible surgical complications associated with this surgery include wound infection, bleeding, dehiscence, flap necrosis, and airway obstruction. These complications are uncommon and were not seen in our series. The average operating time was approximately three hours and free flap reconstruction was avoided in all patients. Donor site morbidity was deemed non-significant (Figs. 2d, 3d, 4e). Patients underwent regular follow-ups without any tumor recurrences noted during this period. Table 2 presents the results obtained in the case of four patients, at least one-year post surgery. Two patients were lost to follow up after the one-year and one-year and eight months visit respectively. All recorded patients had normal lip sensitivity with preservation of facial expressions, speech was fully intelligible and no dietary or cutlery changes were needed. Adequate oral competence was achieved in two patients (sialorrhea with fluid) and complete competence in the other two patients. After surgery, mouth opening was deemed sufficient with all patients being able to insert dentures. Despite slight microstomia being noted, patients reported little to no functional impact as a result. When comparing symmetry of the commissures, only two patients had symmetry at rest. The amount of scaring was visible with proximity in one patient and only during face-to-face discussion in the rest of the patients. Absence of the philtrum was noted only in one case. Overall surgeons and patients were satisfied with functional and aesthetic results. All patients expressed satisfaction with the final facial appearance and were accepting of the scars. Figures 2, 3, 4, 5 and 6 show five patients who were successfully treated using the modified Abbé island flap technique.

## Discussion

Lip reconstruction presents a challenge for reconstructive surgeons and its success requires a strong grasp and understanding of both lip aesthetics and function. While many methods have proved successful for reconstruction of lip defects under 50 % of total width, results and opinions vary for cases when more tissue is lost. This case series presents a new and reliable reconstructive option for over 80 % lip defects following primary lip carcinoma excision: the Modified Abbé Island Flap.

The Abbé flap is a lip switch flap initially described by Robert Abbé M.D. in 1989 [2]. Its use is indicated in lip defects of one-half to two-thirds total length that do not involve the oral commissure. It is an important workhorse in lip reconstruction because it preserves the circumferential orbicularis oris muscles, the vermilion border and natural commissures while re-establishing lip symmetry. Despite the described 65 % defect upper limit of the Abbé flap, our experience shows that, because of its superior functional and aesthetic outcomes compared to free tissue flaps, the technique can be used for larger defects in combination with full thickness or mucosal advancement flaps, such as the Karapandzic flap.

In this cases series, six patients underwent Abbé flap reconstruction combined with bilateral submucosal and/or full thickness cheek advancement. In four patients the advancement of the Karapandzic flap was possible only by using a musculo-mucosal dissection without any incision of the skin. This improved the aesthetic results, however, it was only possible when the laxity of the skin allowed it. Although the size of the defect covered more than 80 % of the lip in all cases, this flap provided adequate stoma size and avoided the use of free flap reconstruction. None of the patients had any functional problems as a result of the minor microstomia. Speech, facial expression, and oral competence were all preserved due to the presence of a functional sphincter and intact innervation. The appearance of the reconstruction was deemed to be acceptable by all our patients one year post-surgery. No wound complications were associated with the procedure, confirming good neo-vascularization of the flap, despite extensive dissection in some cases. Disadvantages reported from this procedure included patient discomfort with regards to a temporary liquid diet and the need for a secondary procedure.

These findings are similar to those reported in other case studies using an Abbé flap for defects less than 65 % of the lip [3–7], although, at the time of writing, we are not aware of any articles describing the combination of Abbé and Karapandzic flaps for more than 80 % lip defects. Our acceptable surgical results support this method as a good alternative to radial forearm flaps for near-total lip defects following tumor resection. Using our combination of the Abbé with the modified Karapandzic flap for reconstruction of lip defects exceeding 80 %, we anticipate reliable mouth opening, avoidance of donor site morbidity and considerable reductions in operative times.

## Conclusion

In conclusion, the head and neck surgeons at McGill University believe that the modified Abbé island flap can be used to reconstruct near-total lip defects using locally innervated, well-vascularized tissues that recreate the oral sphincter and restore oral competence. The combination of the conventional Abbé flap with a modified Karapandzic flap provides reliable results, negligible donor-site morbidity and significantly reduces operating time.

## Abbreviations

BCC, Basal Cell Carcinoma; KA, Keratoacanthoma; MCC, Merkel Cell Carcinoma; SCC, Squamous cell carcinoma
